# Leveraging machine learning to identify optimal patient populations for PEG-IFN therapy in CHB and constructing nomogram models of interferon response: a 48-week follow-up study

**DOI:** 10.1186/s12876-026-04813-6

**Published:** 2026-04-10

**Authors:** Deyang Xi, Ke Xu, Xuebing Yan, Chunyang Li

**Affiliations:** 1 Department of Critical Care Medicine, Xuzhou Central Hospital, Southeast University, Xuzhou, Jiangsu China; 2 Department of Infectious Diseases, The Affiliated Hospital of Xuzhou Medical University, Xuzhou, Jiangsu 221132 China

**Keywords:** CHB, PEG-IFN, Nomogram, Machine learning, Interferon response

## Abstract

**Background and objective:**

Pegylated interferon (PEG-IFN) has been shown to significantly enhance the clinical cure rate in chronic hepatitis B (CHB) patients. This study aims to utilize machine learning to select optimal patient populations for PEG-IFN therapy and construct the nomogram model for predicting interferon response (IR).

**Methods:**

Between April 2020 and October 2023, we collected data from CHB patients treated with PEG-INFα-2b at the Affiliated Hospital of Xuzhou Medical University. Based on HBeAg status, IR is defined as follows: For HBeAg-positive patients, an IR is defined as achieving HBeAg negativity, HBV DNA negativity, and HBsAg levels below 100 IU/mL after 48 weeks of treatment with pegylated interferon; For HBeAg-negative patients, an IR is achieved when HBV DNA becomes undetectable and HBsAg is negative after 48 weeks of pegylated interferon treatment. Through a combination of LASSO COX regression, Random Survival Forests, and CoxBoost, we identified optimal patient populations. A multivariable COX regression was then employed to construct the nomogram model. The performance of the nomogram model was assessed using Receiver Operating Characteristic (ROC) curves, decision curves, and calibration curves.

**Results:**

Utilizing machine learning, we pinpointed baseline HBsAg, △HBsAg, and △ALT as predictors of IR. Leveraging these key predictors, we developed a nomogram model that showcased robust accuracy in predicting IR at 48 weeks across different datasets: the training cohort, validation cohort, and RNA cohort, with AUROC scores of 0.922 (95% CI: 0.88–0.96), 0.938 (95% CI: 0.87–1.00), and 0.933 (95% CI: 0.87–1.00), respectively. The results of the calibration and decision curves show the model has a good consistency and precision. Furthermore, Kaplan–Meier analysis identified that CHB patients with baseline HBsAg < 2.43 lg IU/mL, △HBsAg > 1.26 lg IU/mL, and △ALT > 8.00 U/L were more likely to achieve IR.

**Conclusion:**

The nomogram model can evaluate the efficacy of PEG-IFN in the treatment of CHB patients, and is helpful for clinical decision-making.

**Supplementary Information:**

The online version contains supplementary material available at 10.1186/s12876-026-04813-6.

## Introduction

CHB is a form of chronic liver inflammation caused by HBV infection, serving as a primary cause of chronic, progressive, and end-stage liver diseases [1]. HBV infection represents a significant public health challenge; as of 2019, approximately 296 million individuals worldwide tested positive for the HBsAg, with a global prevalence rate of CHB around 3.5%. Data from the Global Burden of Disease (GBD) study estimates that HBV-related cirrhosis and chronic liver diseases resulted in approximately 331,000 deaths annually [2, 3].

HBV infection is a leading cause of liver fibrosis, cirrhosis, and even hepatocellular carcinoma (HCC) [4]. Currently, the primary treatments for CHB include nucleos(t)ide analogues (NAs) and PEG-IFN [5, 6]. Among these, Pegylated Interferon alpha-2b (PEG-IFNα−2b) is a long-acting interferon with broad-spectrum antiviral and immunomodulatory effects [7], whose production and optimization share common biotechnological foundations with the expression of other antiviral peptides and viral enzymes, such as Shepherin II [8] and the Hepatitis A virus 3C protease [9]**.** Numerous studies have confirmed that treatment with PEG-IFNα−2b for CHB can achieve a higher HBsAg clearance rate compared to NAs, along with a sustained response [10–12]. However, the application of PEG-IFNα−2b in CHB patients has been limited due to its significant side effects, high treatment costs, and considerable controversy over the optimal patient population [13, 14].

Machine learning (ML) exhibits superior capabilities in feature selection, collinearity management, and handling outliers, and has been extensively applied in gene screening, disease diagnosis, and the construction of clinical prognosis models [15, 16]. Variable selection via machine learning can reduce overfitting and enhance model efficiency [17]. In this study, our aim is to utilize machine learning to identify the optimal patient population for PEG-IFNα−2b treatment and construct a nomogram model for predicting interferon response (IR) in CHB patients treated with PEG-IFNα−2b.

## Materials and methods

### Study population

This study retrospectively collected data from CHB patients who received PEG-IFNα−2b treatment at the Affiliated Hospital of Xuzhou Medical University between April 2020 and October 2022. Patients were randomly divided into training and validation cohorts at an 8:2 ratio. Prospectively, from October 2022 to April 2023, CHB patients treated with PEG-IFNα−2b and concurrently tested for HBV RNA at the same hospital were included in the RNA cohort. This study was reviewed and approved by the Ethics Committee of the Affiliated Hospital of Xuzhou Medical University (No: XYFY2021-KL260-01), and informed consent was obtained from all patients.

### Inclusion and exclusion criteria

Patients, characterized by (1) Chronic HBV infection (HBsAg positive for more than 6 months). (2) Age ≥ 18 and ≤ 65 years, regardless of gender. (3) Patients planned or currently undergoing treatment with Pegylated Interferon α−2b, in line with real-world clinical practice needs. (4) Patients who have given informed consent, signed the relevant documents, and complete data. Excluded were (1) individuals with co-infections such as the hepatitis C virus, hepatitis D virus, hepatitis E virus, and human immunodeficiency virus (HIV); (2) presence of liver cirrhosis or hepatocellular carcinoma (HCC); (3) receipt of immunomodulatory therapy within the previous 6 months; (4) discontinuation of treatment before completion of the study protocol; or (5) incomplete clinical data or loss to follow-up. In addition, follow-up visits were required to occur within ± 5 days of the scheduled time point; visits outside this window were missing data.

### Data collection

Patient demographics, baseline characteristics, and serial assessments at 12, 24, and 48 weeks of HBV serological markers, blood routine examination, liver function, and other laboratory tests were systematically collected. Data were categorized into three main types: (1) General information, which includes gender, age, and treatment modality; Based on prior treatment exposure in relation to PEG-IFNα−2b, treatment modality was categorized into two groups. PEG-IFNα−2b INI was defined as patients who had not received any NAs therapy before enrollment and initiated PEG-IFNα−2b monotherapy directly. NAs EXP was defined as patients who had previously received NAs therapy (either as monotherapy or sequential regimens involving lamivudine, entecavir (ETV), telbivudine, adefovir dipivoxil, tenofovir disoproxil fumarate (TDF), or tenofovir alafenamide (TAF)) prior to enrollment and subsequently initiated PEG-IFNα−2b either in combination with ongoing NA therapy or as a switch from prior NAs therapy. (2) Laboratory data encompassing HBV DNA, HBsAg, HBeAg, ALT, AST, GGT, ALP, and PLT; (3) Dynamic indicators comprising changes in HBV DNA (△HBV DNA), HBsAg (△HBsAg), HBeAg (△HBeAg), ALT (△ALT), AST (△AST), GGT (△GGT), ALP (△ALP), and PLT (△PLT) over the initial 12 weeks. Additionally, the RNA cohort underwent HBV RNA testing. All examinations and tests were conducted at the Affiliated Hospital of Xuzhou Medical University. HBV DNA was quantified using the Anpule PCR system (Anpule, China; lower limit of detection: 20 IU/mL). Serum HBsAg and HBeAg were measured by Roche Elecsys assays (Roche, Switzerland), with lower limits of detection of 0.03 IU/mL for HBsAg and 1.00 COI for HBeAg. HBV RNA was detected using the RNA capture probe assay (Rendu Biotechnology, China), with lower limits of detection of 50 copies/mL. Liver function parameters, including AST, ALT, GGT, and ALP, were measured using Roche diagnostics, with reference ranges of 15–40 U/L, 9–50 U/L, 10–60 U/L, and 42–128 U/L, respectively. PLT count was determined using an automated hematology analyzer.

### Interferon response (IR) definition

PEG-IFNα−2b used in this study was Pegbin (Xiamen Amoytop Biotech Co., Ltd., Xiamen, China). The drug was administered by subcutaneous injection at a dose of 180 μg once weekly. According to the study protocol, the standard treatment duration for all patients was 48 weeks.

For HBeAg-positive patients, an IR is defined as achieving HBeAg negativity, HBV DNA negativity, and HBsAg levels below 100 IU/mL after 48 weeks of treatment with pegylated interferon; For HBeAg-negative patients, an IR is achieved when HBV DNA becomes undetectable and HBsAg is negative after 48 weeks of pegylated interferon treatment.

Patients are categorized into two groups based on their status at the 48-week endpoint: those who achieve an interferon response (IR group) and those who do not interferon response (N-IR group).

### Machine learning

Variable selection via machine learning can mitigate overfitting and enhance the efficiency of models. Binary COX regression models, known for their strong interpretability, are particularly suited for classification problems [18]. The integration of machine learning with COX regression models facilitates the creation of models that are both accurate and interpretable. In this study, we employed the LASSO COX regression model to identify predictors associated with the response variable. To determine the optimal regularization strength (λ), we applied a tenfold cross-validation method, selecting the lambda.min (0.021) that minimized the cross-validation error as the optimal regularization parameter. Based on this parameter, the LASSO model was trained, retaining only those predictors with non-zero coefficients, thereby achieving variable selection and shrinkage. We explored significant variables in the data using the Random Survival Forest (RSF) method. In optimizing the RSF model parameters, we identified an optimal node size of 3 and the number of features to consider at each split (Mtry) as 7. Based on these optimal parameters, we built a final RSF model with 30 trees. By assessing the importance scores of variables, we filtered those predictors with coefficients greater than 0 as significant variables. The CoxBoost model was applied, determining the optimal step as 61 steps through tenfold cross-validation combined with a penalty strength of 500, and selecting variables with non-zero coefficients. Finally, a multivariable COX regression model was constructed.

### Statistical analysis

Statistical analyses in this study were conducted using R software version 4.3.1. Quantitative data conforming to a normal distribution were presented as mean (standard deviation), and comparisons between two groups were made using the paired t-test. Non-normally distributed quantitative data were presented as median [P25; P75], and comparisons between groups were conducted using the Wilcoxon rank-sum test. Categorical variables between two groups were compared using the χ2 test. Variable selection was performed using LASSO COX regression, Random Survival Forest, and CoxBoost machine learning methods. A multivariable COX regression analysis was used to construct the nomogram model, and its predictive ability was evaluated using ROC curve analysis, comparing predictive values based on the area under the ROC curve (AUC). Calibration curves and decision curves were drawn to assess the model's consistency and precision. The “survminer” R package's “surv_cutpoint” function, based on the maximal selected rank statistics method, was used to find the optimal threshold for delineating the target population. Kaplan–Meier (K-M) curves were employed to display the cumulative incidence of events between two groups.

## Results

### Patient characteristics

**Fig. 1:**
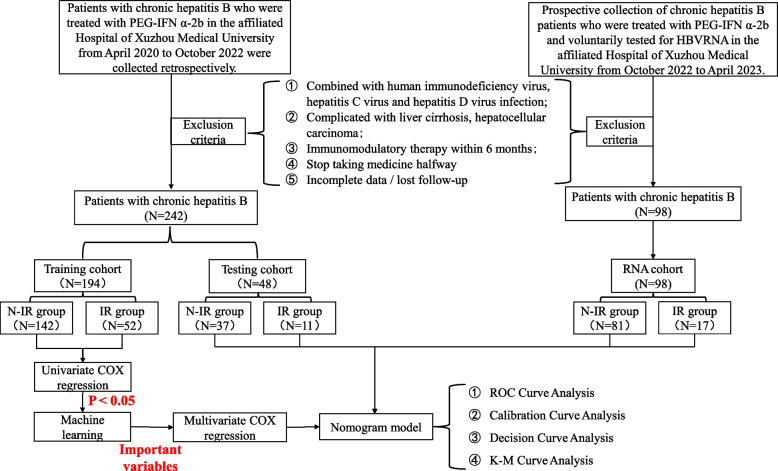
Flow diagram of the study

Figure 1 illustrates the technical roadmap of this study. We retrospectively collected data from CHB patients who visited the Affiliated Hospital of Xuzhou Medical University between April 2020 and October 2022. Based on the inclusion and exclusion criteria, a total of 242 CHB patients were enrolled. These patients were randomly divided into a training cohort of 194 patients and a validation cohort of 48 patients at an 8:2 ratio. Additionally, a prospective RNA cohort of 98 patients was collected from October 2022 to April 2023, who underwent HBV RNA testing. The validation cohort and RNA cohort served as validation sets. Table 1 presents baseline data for patients with an IR and N-IR. Differences were observed between the two groups in treatment method, baseline HBV RNA, baseline HBsAg, baseline HBeAg, △HBsAg, △HBVDNA, baseline AST, baseline ALT, baseline GGT, baseline ALP, △AST, △ALT, △GGT, △ALP, and △PLT. In patients who achieved an IR, virological markers (HBsAg, HBV DNA, HBeAg) were lower, and liver damage indicators (AST, ALT, GGT, ALP) were higher than in the N-IR group. Table S1 shows baseline characteristics for the training and validation cohorts of CHB patients, indicating no statistical differences between the indicators of the two cohorts.

**Table 1:** Comparison of clinical data between IR group and N-IR group

	**N-IR group**	**IR group**	**Statistical value**	***P* value**
	***N* = *179***	***N* = *63***		
Gender:			*X*^*2*^ = 0.615	0.433
Male	126 (70.4%)	41 (65.1%)		
Female	53 (29.6%)	22 (34.9%)		
Age:	38.2 (8.84)	39.5 (7.92)	*t* = −1.100	0.273
Treatment:			*X*^*2*^ = 5.409	0.020
PEG-IFNα−2b INI	75 (41.9%)	16 (25.4%)		
NAs EXP	104 (58.1%)	47 (74.6%)		
Baseline HBV DNA:			*X*^*2*^ = 7.613	0.009
Negative	92 (51.4%)	45 (71.4%)		
Positive	87 (48.6%)	18 (28.6%)		
Baseline HBsAg (lg IU/mL)	3.10 [2.86;3.51]	2.51 [2.07;2.86]	*Z* = −7.934	< 0.001
Baseline HBeAg:			*X*^*2*^ = 6.832	0.014
Negative	97 (54.2%)	46 (73.0%)		
Positive	82 (45.8%)	17 (27.0%)		
△HBsAg (lg IU/mL)	0.14 [0.01;0.42]	1.04 [0.48;1.70]	*Z* = −6.911	< 0.001
△HBeAg (lg COI)	0.13 [0.00; 0.40]	0.17 [0.00;0.79]	*Z* = −0.708	0.479
△HBVDNA (lg IU/mL)	0.00 [0.00;2.09]	0.00 [0.00;0.32]	*Z* = −2.545	0.011
Baseline AST (U/L)	23.0 [19.0;34.5]	20.0 [16.0;25.5]	*Z* = −3.394	0.001
Baseline ALT (U/L)	30.0 [19.0;57.5]	19.0 [15.0;28.5]	*Z* = −4.163	< 0.001
Baseline GGT (U/L)	23.0 [16.0;35.0]	20.0 [13.5;30.0]	*Z* = −1.987	0.047
Baseline ALP (U/L)	75.3 (32.8)	68.5 (17.7)	*t* = 2.052	0.042
△AST (U/L)	16.0 [1.50; 32.0]	31.0 [19.5; 53.0]	*Z* = −4.574	< 0.001
△ALT (U/L)	−17.00 [−42.50; 9.50]	−36.00 [−64.00; −23.50]	*Z* = −4.660	< 0.001
△GGT (U/L)	23.0 [9.00;47.5]	33.0 [18.5;59.5]	*Z* = −2.253	0.024
△ALP (U/L)	4.52 (16.6)	8.73 (13.5)	*t* = −1.997	0.048
Baseline PLT(10^9/L)	195 (61.2)	195 (63.4)	*t* = −0.044	0.965
△PLT(10^9/L)	−63.23 (64.8)	−81.89 (56.5)	*t* = 2.168	0.032

### Screening important variables by machine learning

**Fig. 2:**
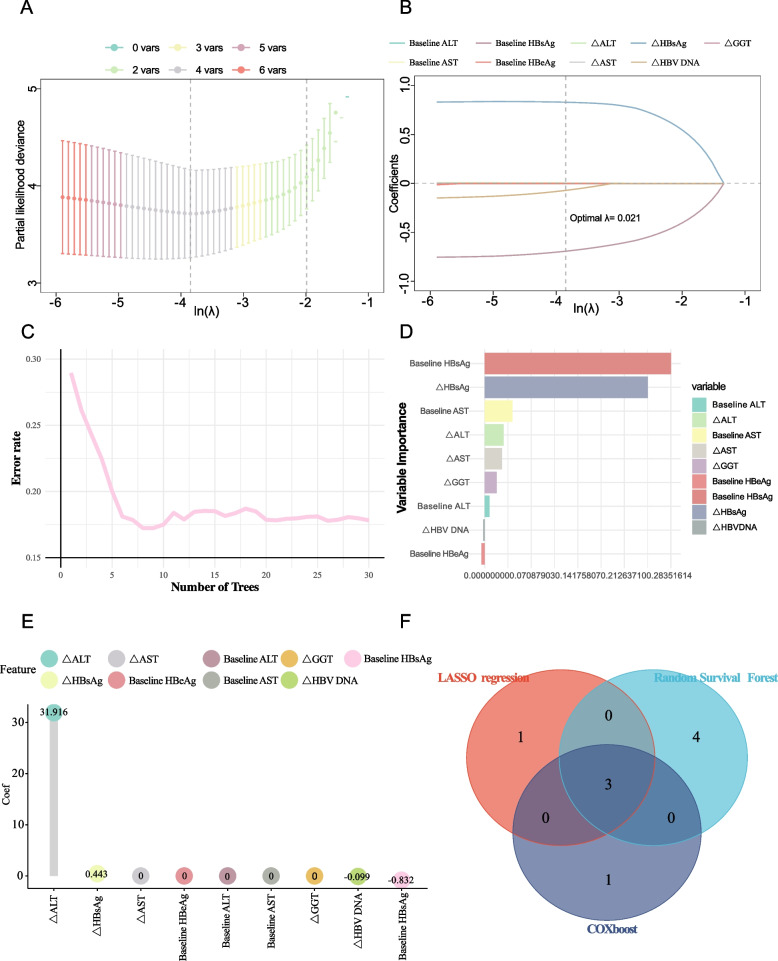
Screening variables for predicting IR using machine learning algorithms. LASSO COX regression Curv (**A**) and Cross-validation plot (**B**). Random Survival Forest Analysis illustrating the error rate as the number of trees increases (**C**) and the importance of each variable in the model (**D**). CoxBoost Analysis highlighting the variables with non-zero coefficients deemed important (**E**). Venn Diagram showing the intersection of selected variables across the three machine learning methods (**F**)

Incorporating a total of 19 variables, encompassing general information, laboratory data, and dynamic indicators into a univariate COX regression analysis, it was found that baseline HBsAg, baseline HBeAg, △HBsAg, △HBVDNA, baseline AST, baseline ALT, △AST, △ALT, and △GGT exhibited statistically significant differences (Table S2). These 9 variables were further scrutinized through machine learning for variable selection. LASSO COX regression with tenfold cross-validation was applied, using the cross-validation λ min value (λ = 0.021) for variable selection, resulting in LASSO COX regression curves and cross-validation plots (Fig. 2A-B). Based on LASSO COX regression, 4 variables with non-zero coefficients were identified as significant: baseline HBsAg, △HBsAg, △HBVDNA, and △ALT. Random Survival Forest (RSF) selected 7 important variables: baseline HBsAg, △HBsAg, baseline AST, baseline ALT, △ALT, △AST, and △GGT (Fig. 2C-D). The CoxBoost algorithm identified 4 non-zero significant variables: baseline HBsAg, △HBsAg, △HBV DNA, and △ALT (Fig. 2E). By intersecting the findings from the three machine learning algorithms (Fig. 2F), baseline HBsAg, △HBsAg, and △ALT were determined to be the most critical variables for predicting IR.

### Construction of the nomogram model

**Fig. 3:**
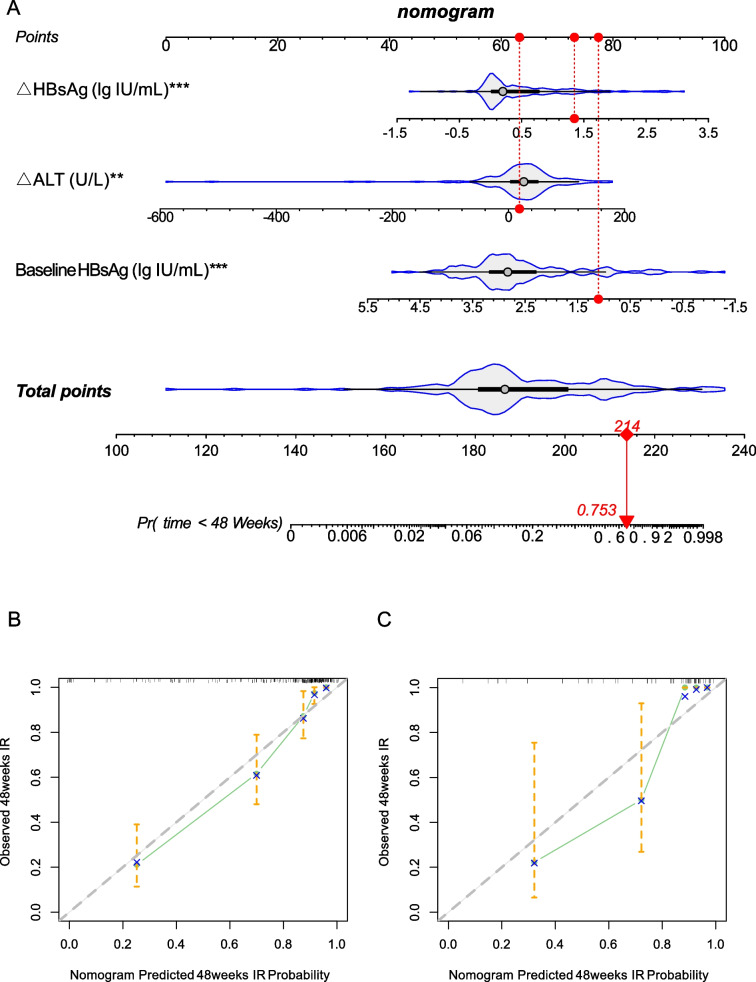
Construction of the nomogram model (**A**) for Predicting IR and Calibration Curve Analysis of the nomogram model in the training cohort (**B**) and validation cohort (**C**)

To enhance the interpretability of the model, a multivariable COX regression analysis was conducted, incorporating the three crucial variables identified through machine learning. The study revealed that baseline HBsAg, △HBsAg, and △ALT are independent predictors of achieving an IR in CHB patients (Table 2). To improve the visualization of the model's findings, a nomogram was constructed (Fig. 3A). This graphical representation allows for the straightforward prediction of IR based on the values of these significant predictors, thereby providing a useful tool for clinicians in the decision-making process for treating CHB patients.

**Table 2:** Multivariate COX regression analysis for predicting IR

variables	Multivariate COX regression		
	HR	95%CI	P
Baseline HBsAg (lg IU/mL):	0.44	0.35–0.56	< 0.001
△HBsAg (lg IU/mL):	2.65	1.90–3.70	< 0.001
△ALT (U/L):	1.01	1.00–1.02	0.004

### Prediction performance of calibration curve and decision curve evaluation model

**Fig. 4:**
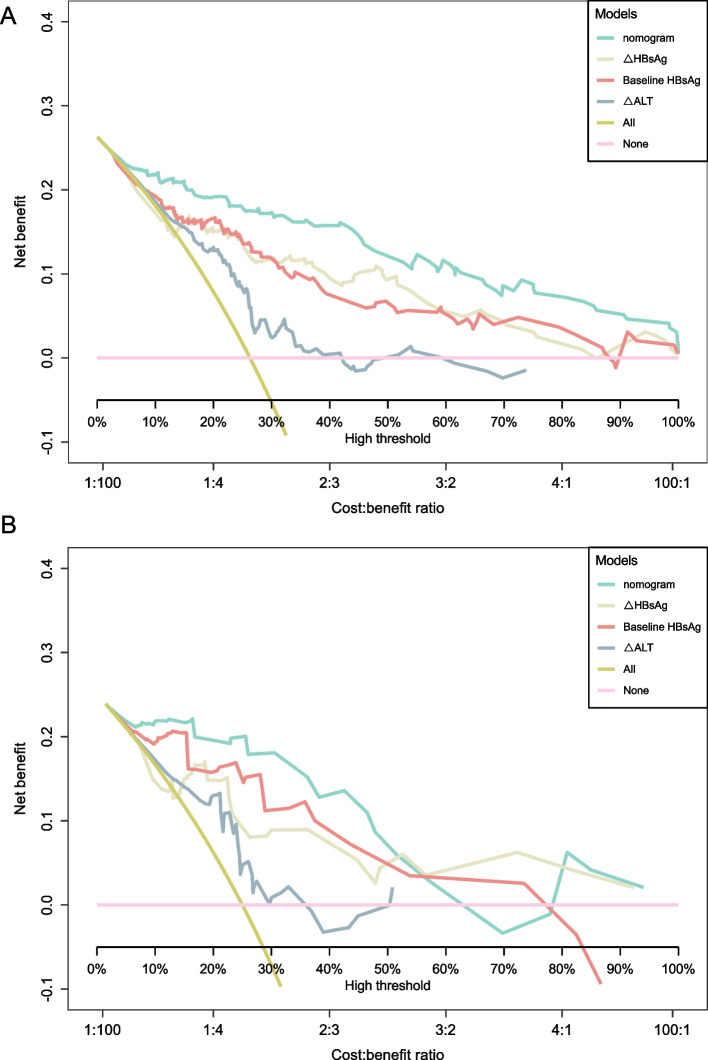
Decision Curve Analysis of the nomogram model in the training cohort (**A**) and validation cohort (**B**)

To validate the accuracy of the constructed nomogram model, calibration and decision curves were plotted. The calibration curves (Fig. 3B-C) demonstrate that the predicted probabilities of the nomogram model closely align with the actual probabilities. The decision curve analysis (Fig. 4A-B) indicates that the nomogram model provides consistent net benefits within a certain threshold probability range and surpasses the independent predictors in forecasting the IR. To further evaluate the predictive performance of the nomogram model across different HBV DNA and HBeAg status subgroups, we combined the training and validation cohorts (total *n* = 242) and conducted subgroup analyses. The results demonstrated that the nomogram model consistently exhibited strong predictive value for IR across all subgroups (Figure S1), with AUC values all exceeding 0.88 (Figure S2).

### Screening the dominant population for PEG-IFN in treating CHB

**Fig. 5:**
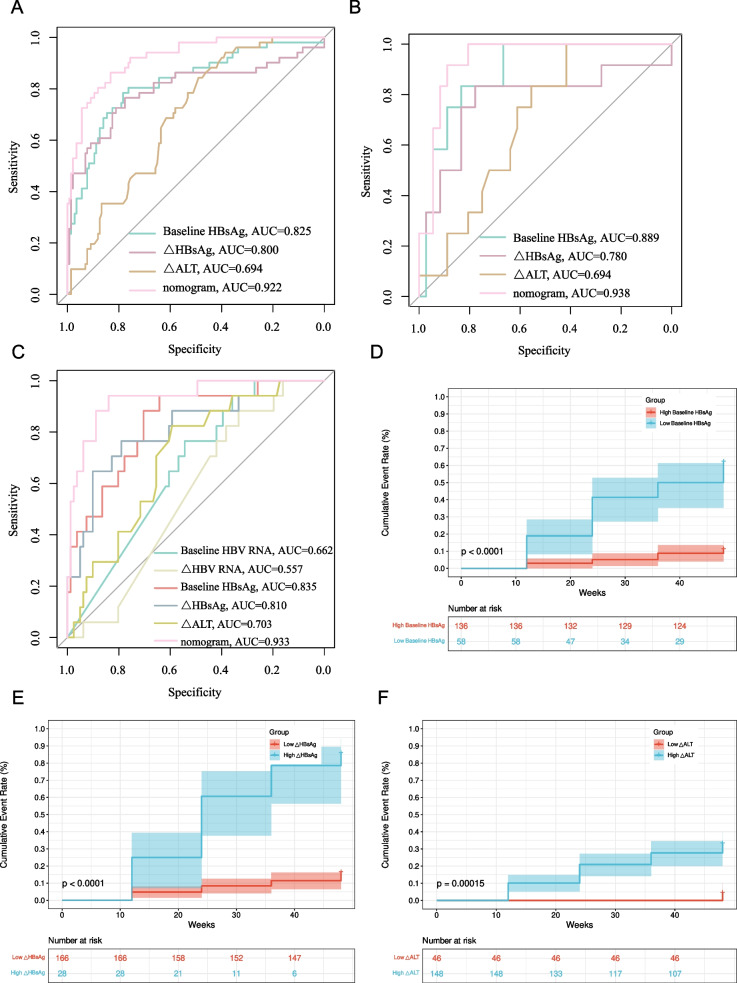
AUROC of Independent Impact Factors and Nomogram Model in the Training Cohort (**A**), Validation Cohort (**B**), and RNA Cohort (**C**), along with Kaplan–Meier Curve Analysis of Baseline HBsAg (**D**), △HBsAg (**E**), and △ALT (**F**)

To further validate the accuracy of the model, we prospectively collected an independent RNA cohort comprising 98 patients, including 81 in the N-IR group and 17 in the IR group. The clinical characteristics of the RNA cohort are presented in Table S3. Baseline clinical characteristics were well balanced among the training set, validation set, and RNA cohort (Table S1). In terms of predicting IR, the nomogram model we constructed demonstrated high predictive capability with AUROC of 0.922 in the training cohort, 0.938 in the validation cohort, and 0.933 in the RNA cohort (Fig. 5A-C). Interestingly, in the RNA cohort, where we also tested patients' virological markers alongside HBV RNA, our nomogram model showed a higher predictive value for IR compared to virological markers and HBV RNA alone (Table S4). To further investigate the patient subgroup most likely to benefit from IR, Kaplan–Meier (KM) curve analyses were conducted for the independent predictors: baseline HBsAg, △HBsAg, and △ALT (Fig. 5D-F). The results indicated that patients with a low baseline level of HBsAg (baseline HBsAg < 2.43 lg IU/mL), a high change in △HBsAg (△HBsAg > 1.26 lg IU/mL), and a high change in △ALT (△ALT > 8.00 U/L) were more likely to achieve an IR. Consequently, patients characterized by baseline HBsAg < 2.43 lg IU/mL, △HBsAg > 1.26 lg IU/mL, and △ALT > 8.00 U/L were considered as the subgroup most likely to benefit from interferon therapy.

## Discussion

In this study, we utilized three machine learning techniques to identify key predictors of IR and developed and validated a nomogram model to predict the response to pegylated interferon treatment in CHB patients. This model demonstrated high accuracy in the training cohort, validation cohort, and RNA cohort. Additionally, we assessed the net benefit and diagnostic performance of the nomogram model by drawing ROC curves, decision curves, and calibration curves. Our research is of significant importance for clinicians in evaluating the effectiveness of pegylated interferon treatment in CHB patients and in adjusting medication.

PEG IFN possesses the dual capability of inhibiting viral replication and activating immune responses, capable of inducing the proliferation of cytotoxic T cells, regulating immune pathways, and reducing the levels of covalently closed circular DNA (cccDNA), thereby clearing HBV from infected cells [19–21]. Numerous studies have confirmed that optimal patients undergoing treatment based on PEG IFNα can achieve a clinical cure rate of 30%−80% [22–24]. Many studies [25–27] have shown that patients with lower levels of HBsAg, and/or lower levels of HBeAg and HBV DNA, and those who experience significant reductions in HBsAg during treatment, are more likely to achieve clinical cure. However, there is still some controversy over the specific cut-off values for each indicator and the quantification of IR through nomogram prediction models. Our study, through machine learning-selected optimal populations and the construction of a nomogram model, provides a tool for real-time dynamic monitoring of the efficacy of PEG IFN treatment, to some extent, compensating for the quantitative inadequacies. Consistent with prior research, we consider CHB patients with baseline HBsAg < 2.43 lg IU/mL, △HBsAg > 1.26 lg IU/mL, and △ALT > 8.00 U/L as the optimal population, offering quantified cutoff values.

Baseline HBsAg levels partially reflect the transcriptional activity of intrahepatic cccDNA as well as the expression of integrated HBV DNA, and therefore serve as an important indicator of viral replication status and host immune control [28]. Previous studies have shown that baseline HBsAg possesses relatively good specificity in predicting PEG-IFN–induced HBsAg clearance [29]. Accordingly, baseline HBsAg can be used as an important criterion for pre-treatment patient stratification [30]. In patients with relatively high HBsAg levels (e.g., > 3.4 log10 IU/mL), the likelihood of achieving HBsAg clearance with PEG-IFN is markedly reduced [31]. In contrast, the dynamic kinetics of HBsAg, particularly the magnitude of its early decline during therapy, has been widely recognized as one of the most informative biomarkers for predicting PEG-IFN treatment response [32]. Multiple clinical cohort studies and meta-analyses have demonstrated that a rapid decline in HBsAg during the early phase of treatment (especially within 12–24 weeks) is strongly associated with subsequent HBsAg clearance, HBeAg seroconversion, and functional cure. For instance, a reduction of ≥ 1 log10 IU/mL in HBsAg at week 12 has been shown to significantly increase the probability of HBsAg clearance [30]. Mechanistically, PEG IFN induces the expression of interferon-stimulated genes (ISGs), which suppress cccDNA transcription while simultaneously enhancing HBV-specific immune responses [33]. Consequently, an early decline in HBsAg generally reflects effective suppression of viral replication together with progressive immune-mediated clearance of infected hepatocytes. Moreover, the reduction in circulating HBsAg antigen burden may alleviate HBV-specific T-cell exhaustion, thereby facilitating the restoration of host immune function and strengthening immune-mediated viral control [34]. Consistent with these observations, our findings indicate that patients with HBsAg < 2.43 log IU/mL and ΔHBsAg > 1.26 log IU/mL are more likely to achieve an IR. Interestingly, a mild increase in ALT during 0–12 weeks and > 8.00 U/L appears to aid in achieving an IR. This could be due to the immune activation triggered by HBV infection, which might affect T cells and B cells, causing liver cell damage and thus an increase in ALT [35]. HBV RNA, as a novel marker of chronic hepatitis B virus infection, derived solely from cccDNA and well-correlated with other serological viral markers, is considered an ideal serological surrogate marker for liver tissue cccDNA [36]. Our study in the RNA cohort explored the capability of our constructed nomogram model and virological indicators, including HBV RNA, in predicting the IR. However, our study has certain limitations. Firstly, it is a single-center study with a relatively small sample size, necessitating validation in multicenter studies with larger external cohorts. Secondly, our research focused exclusively on patients undergoing treatment with pegylated interferon. The predictive efficacy for CHB patients treated with NAs requires further investigation in future studies.

In summary, utilizing machine learning, we identified the optimal patient population for pegylated interferon treatment in CHB and constructed a nomogram model to predict IR. Furthermore, analyses using ROC curves, decision curves, and calibration curves have confirmed the feasibility of our model in predicting IR.

## Supplementary Information

Supplementary Material 1. Table S1. Comparison of clinical data between training cohort, testing cohort and RNA cohort. Table S2. Univariate COX regression analysis for predicting IR. Table S3. Comparison of clinical data between IR group and N-IR group in RNA cohort. Table S4. AUROC and 95% CI of Independent Impact Factors and Nomogram in the Training Cohort, Validation Cohort, and RNA Cohort. Figure S1. Forest plot of subgroup analyses for the nomogram model. Figure S2. ROC curve analysis of the nomogram model in subgroups defined by HBV DNA and HBeAg status.

## Data Availability

No additional data are available. For additional inquiries, please contact the author, Chunyang Li, at [13775989791@126.com] (mailto:13775989791@126.com).

## Abbreviations

ALP: Alkaline phosphatase
ALT: Alanine aminotransferase
AST: Aspartate aminotransferase
AUC: Area under the curve
AUROC: Area under the receiver operating characteristic curve
cccDNA: Covalently closed circular DNA
CHB: Chronic hepatitis B
CI: Confidence interval
CLDs: Chronic liver diseases
ETV: Entecavir
GBD: Global Burden of Disease
GGT: Gamma-glutamyl transferase
HBsAg: Hepatitis B surface antigen
HBeAg: Hepatitis B e antigen
HBV: Hepatitis B virus
HBV DNA: Hepatitis B virus DNA
HBV RNA: Hepatitis B virus RNA
HCC: Hepatocellular carcinoma
HR: Hazard ratio
IR: Interferon response
KM: Kaplan–Meier
LASSO: Least absolute shrinkage and selection operator
ML: Machine learning
NAs: Nucleos(t)ide analogues
N-IR: Non-interferon response
PEG-IFN: Pegylated interferon
PLT: Platelet count
ROC: Receiver operating characteristic
RSF: Random survival forest
TDF: Tenofovir disoproxil fumarate
TAF: Tenofovir alafenamide
