# Supplementary material for: Leveraging machine learning to identify optimal patient populations for PEG-IFN therapy in CHB and constructing nomogram models of interferon response: a 48-week follow-up study
Source: BMC Gastroenterol. 2026 Apr 10;26:348. doi: 10.1186/s12876-026-04813-6 (PMC13237905; doi:10.1186/s12876-026-04813-6)

**Table S1.** Comparison of clinical data between Training cohort, Validation cohort and RNA cohort.

|  | **Training cohort** | **Validation cohort** | **RNA cohort** | **P value** |
| --- | --- | --- | --- | --- |
|  | ***N=194*** | ***N=48*** | ***N=98*** |  |
| Gender: |  |  |  | 0.296 |
| Male | 130 (67.0%) | 37 (77.1%) | 60 (61.2%) |  |
| Female | 64 (33.0%) | 11 (22.9%) | 38 (38.8%) |  |
| Age： | 39.0 [33.0;45.0] | 37.5 [33.0;44.2] | 39.0 [34.0;44.0] | 0.960 |
| Treatment: |  |  |  | 0.006 |
| PEG-IFNα-2b INI | 72 (37.1%) | 19 (39.6%) | 55 (56.1%) |  |
| NAs EXP | 122 (62.9%) | 29 (60.4%) | 43 (43.9%) |  |
| Baseline HBV DNA: |  |  |  | 0.100 |
| Negative | 111 (57.2%) | 26 (54.2%) | 43 (43.9%) |  |
| Positive | 83 (42.8%) | 22 (45.8%) | 55 (56.1%) |  |
| Baseline HBsAg (lg IU/mL) | 2.83 [2.29;3.18] | 2.63 [1.74;3.14] | 2.83 [1.94;3.24] | 0.887 |
| Baseline HBeAg: |  |  |  | 0.024 |
| Negative | 116 (59.8%) | 27 (56.2%) | 72 (73.5%) |  |
| Positive | 78 (40.2%) | 21 (43.8%) | 26 (26.5%) |  |
| △HBsAg (lg IU/mL) | 0.20 [0.02;0.78] | 0.26 [0.04;0.82] | 0.36 [0.09;1.11] | 0.173 |
| △HBeAg (lg COI) | 0.13 [0.00;0.47] | 0.07 [-0.05;0.32] | 0.10 [0.00;0.56] | 0.726 |
| △HBVDNA (lg IU/mL) | 0.00 [0.00;1.89] | 0.00 [0.00;1.99] | 0.29 [0.00;1.58] | 0.549 |
| Baseline AST (U/L) | 22.0 [17.2;29.0] | 24.0 [18.8;46.0] | 22.0 [18.0;26.8] | 0.141 |
| Baseline ALT (U/L) | 26.0 [17.0;46.0] | 25.0 [18.8;64.8] | 24.0 [17.0;36.0] | 0.082 |
| Baseline GGT (U/L) | 21.0 [15.0;34.0] | 23.0 [15.5;33.2] | 18.0 [14.0;29.2] | 0.140 |
| Baseline ALP (U/L) | 69.0 [57.2;83.0] | 68.5 [62.0;80.5] | 69.0 [60.2;85.0] | 0.681 |
| △AST (U/L) | 21.0 [7.25;42.8] | 15.5 [-2.25;29.8] | 22.5 [10.2;45.0] | 0.223 |
| △ALT (U/L) | -26.50 [-50.75; -4.00] | -16.50 [-40.25;11.2] | 28.5 [13.0;53.2] | 0.227 |
| △GGT (U/L) | 27.0 [11.2;51.0] | 22.5 [8.75;53.2] | 22.0 [10.2;54.8] | 0.742 |
| △ALP (U/L) | 6.34 (14.9) | 2.69 (19.6) | 10.5 (20.6) | 0.067 |
| Baseline PLT | 196 (62.4) | 189 (58.6) | 196 (61.8) | 0.957 |
| △PLT | -73.00 [-113.75; -31.50] | -65.00 [-92.00; -25.00] | -77.50 [-114.00; -39.50] | 0.126 |

**Table S2.** Univariate COX regression analysis for predicting IR

| variables | Univariate COX regression | | |
| --- | --- | --- | --- |
|  | HR | 95%CI | P |
| Gender: | 1.25 | 0.71-2.20 | 0.446 |
| Age： | 1.01 | 0.97-1.04 | 0.703 |
| Treatment: | 1.71 | 0.92-3.16 | 0.089 |
| Baseline HBV DNA: | 0.55 | 0.37-1.52 | 0.060 |
| Baseline HBsAg (lg IU/mL) | 0.41 | 0.33-0.51 | <0.001 |
| Baseline HBeAg: | 0.45 | 0.24-0.85 | 0.013 |
| △HBsAg (lg IU/mL): | 3.36 | 2.50-4.52 | <0.001 |
| △HBeAg (lg COI): | 1.25 | 0.90-1.75 | 0.183 |
| △HBVDNA (lg IU/mL): | 0.77 | 0.62-0.97 | 0.027 |
| Baseline AST (U/L): | 0.97 | 0.95-1.00 | 0.027 |
| Baseline ALT (U/L): | 0.98 | 0.97-1.00 | 0.014 |
| Baseline GGT (U/L): | 0.99 | 0.97-1.00 | 0.104 |
| Baseline ALP (U/L): | 0.99 | 0.97-1.00 | 0.111 |
| △AST (U/L): | 1.01 | 1.00-1.01 | 0.001 |
| △ALT (U/L): | 1.01 | 1.01-1.02 | <0.001 |
| △GGT (U/L): | 1.01 | 1.00-1.01 | 0.038 |
| △ALP (U/L): | 1.01 | 1.00-1.03 | 0.128 |
| Baseline PLT: | 1.00 | 0.99-1.00 | 0.387 |
| △PLT: | 1.00 | 0.99-1.00 | 0.265 |

**Table S3.** Comparison of clinical data between IR group and N-IR group in RNA cohort

|  | **N-IR group** | **IR group** | **Statistical** | **P value** |
| --- | --- | --- | --- | --- |
|  | ***N=81*** | ***N=17*** | **value** |  |
| Gender: |  |  | *X^2^*= 0.105 | 0.745 |
| Male | 32 (39.5%) | 6 (35.3%) |  |  |
| Female | 49 (60.5%) | 11 (64.7%) |  |  |
| Age： | 38.9 (8.67) | 39.5 (6.61) | *t* = -0.349 | 0.730 |
| Treatment: |  |  | *X^2^*= 0.686 | 0.407 |
| PEG-IFNα-2b INI | 47 (58.0%) | 8 (47.1%) |  |  |
| NAs EXP | 34 (42.0%) | 9 (52.9%) |  |  |
| Baseline HBV RNA: |  |  | *X^2^*=2.438 | 0.118 |
| Negative | 31 (38.3%) | 10 (58.8%) |  |  |
| Positive | 50 (61.7%) | 7 (41.2%) |  |  |
| Baseline HBV DNA: |  |  | *X^2^*= 1.866 | 0.172 |
| Negative | 33 (40.7%) | 10 (58.8%) |  |  |
| Positive | 48 (59.3%) | 7 (41.2%) |  |  |
| Baseline HBsAg (lg IU/mL) | 2.97 [2.39;3.31] | 1.65 [-0.09;2.41] | *Z* = -7.934 | <0.001 |
| Baseline HBeAg: |  |  | *X^2^*= 6.832 | 0.014 |
| Negative | 61 (75.3%) | 11 (64.7%) |  |  |
| Positive | 20 (24.7%) | 6 (35.3%) |  |  |
| △HBV RNA (lg copies/mL) | 0.00 [0.00;0.75] | 0.00 [0.00;0.14] | *Z* =-0.761 | 0.450 |
| △*HBsAg (lg IU/mL) | 0.29 [0.04;0.72] | 1.45 [0.82;2.04] | *Z* = -4.330 | <0.001 |
| △HBeAg (lg COI) | 0.29 (0.75) | 0.24 (0.40) | *t* = -0.371 | 0.712 |
| △HBVDNA (lg IU/mL) | 0.48 [0.00;1.60] | 0.00 [0.00;0.70] | *Z* = -1.370 | 0.171 |
| Baseline AST (U/L) | 22.0 [18.0;28.0] | 20.0 [18.0;24.0] | *Z* = -1.100 | 0.271 |
| Baseline ALT (U/L) | 24.0 [17.0;38.0] | 19.0 [15.0;27.0] | *Z* = -1.568 | 0.117 |
| Baseline GGT (U/L) | 18.0 [14.0;32.0] | 20.0 [15.0;22.0] | *Z* = -0.052 | 0.959 |
| Baseline ALP (U/L) | 75.4 (25.6) | 67.4 (19.6) | *t* = 1.439 | 0.161 |
| △AST (U/L) | 21.0 [9.00;44.0] | 31.0 [22.0;68.0] | *Z* = -1.708 | 0.088 |
| △ALT (U/L) | 25.0 [9.00;46.0] | 44.0 [33.0;91.0] | *Z* = -2.618 | 0.009 |
| △GGT (U/L) | 22.0 [9.00;47.0] | 22.0 [18.0;84.0] | *Z* = -1.375 | 0.169 |
| △ALP (U/L) | 10.3 (21.4) | 11.2 (17.4) | *t* = -1.862 | 0.854 |
| Baseline PLT | 194 (59.2) | 204 (74.3) | *t* = -0.556 | 0.584 |
| △PLT | -67.77 (64.3) | -99.00 (73.4) | *t* =1.628 | 0.118 |

|  | Training Cohort | | Validation Cohort | | RNA Cohort | |
| --- | --- | --- | --- | --- | --- | --- |
|  | AUROC | 95%CI | AUROC | 95%CI | AUROC | 95%CI |
| Nomogram | 0.922 | 0.88-0.96 | 0.938 | 0.87-1.00 | 0.933 | 0.87-1.00 |
| Baseline HBsAg (lg IU/mL) | 0.825 | 0.75-0.90 | 0.889 | 0.79-0.98 | 0.835 | 0.73-0.94 |
| △HBsAg (lg IU/mL) | 0.800 | 0.71-0.88 | 0.780 | 0.59-0.97 | 0.810 | 0.69-0.93 |
| △ALT (U/L) | 0.694 | 0.62-0.77 | 0.694 | 0.54-0.85 | 0.703 | 0.58-0.83 |
| Baseline HBV RNA (lg copies/mL) | NA | NA | NA | NA | 0.662 | 0.55-0.78 |
| △HBV RNA (lg copies/mL) | NA | NA | NA | NA | 0.557 | 0.44-0.68 |

**Table S4.** AUROC and 95% CI of Independent Impact Factors and Nomogram in the Training Cohort, Validation Cohort, and RNA Cohort

**Figure S1.** Forest plot of subgroup analyses for the nomogram model.


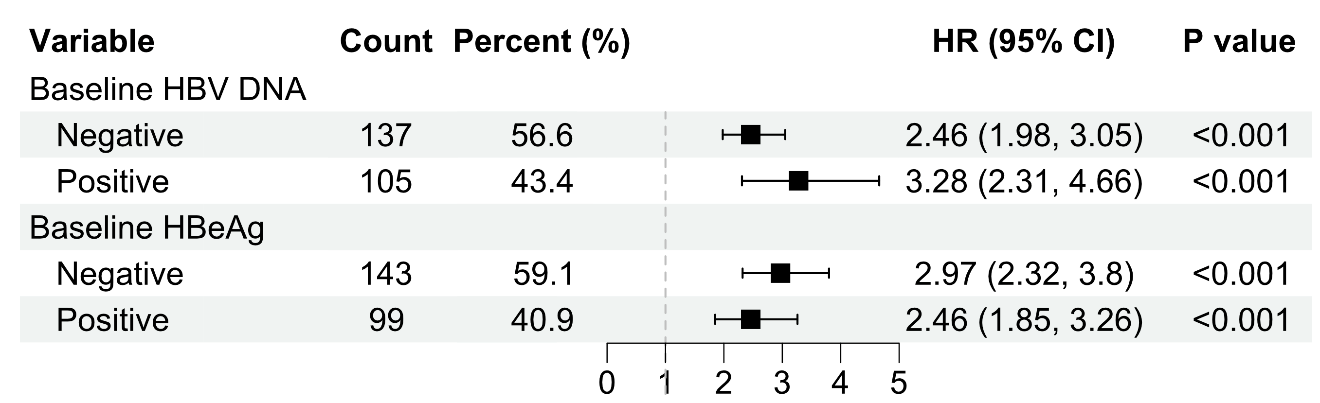


**Figure S2**. ROC curve analysis of the nomogram model in subgroups defined by HBV DNA and HBeAg status.


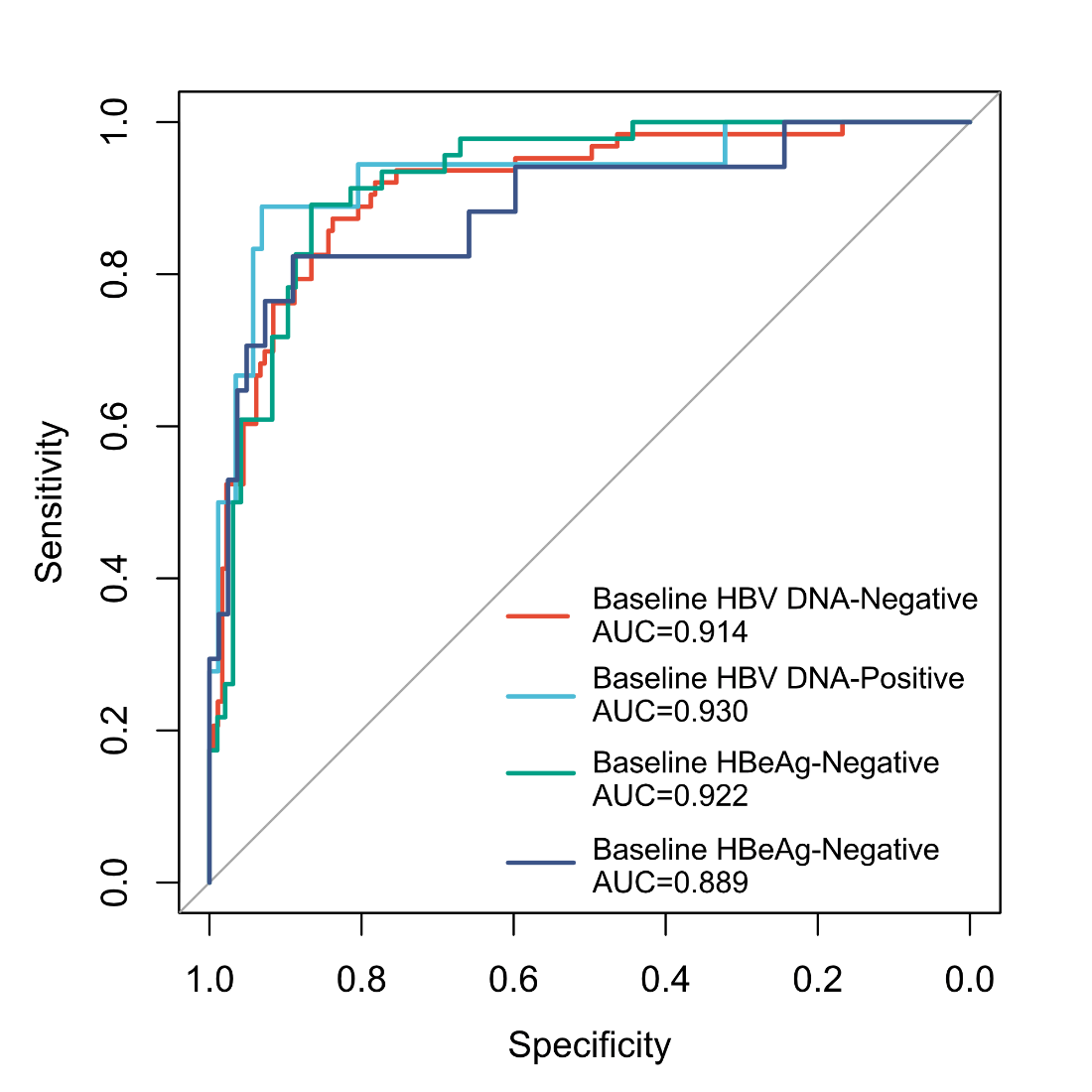

Supplement: Supplementary file 1 — Supplementary Material 1. Table S1. Comparison of clinical data between training cohort, testing cohort and RNA cohort. Table S2. Univariate COX regression analysis for predicting IR. Table S3. Comparison of clinical data between IR group and N-IR group in RNA cohort. Table S4. AUROC and 95% CI of Independent Impact Factors and Nomogram in the Training Cohort, Validation Cohort, and RNA Cohort. Figure S1. Forest plot of subgroup analyses for the nomogram model. Figure S2. ROC curve analysis of the nomogram model in subgroups defined by HBV DNA and HBeAg status. [file 12876_2026_4813_MOESM1_ESM.docx]
